# Supplementary material for: Uncovering PheCLE1 and PheCLE10 Promoting Root Development Based on Genome-Wide Analysis
Source: Int J Mol Sci. 2024 Jun 29;25(13):7190. doi: 10.3390/ijms25137190 (PMC11241622; doi:10.3390/ijms25137190)
Supplement: Supplementary file 1 [file ijms-25-07190-s001.zip › Supplemental Figure S1.pdf]

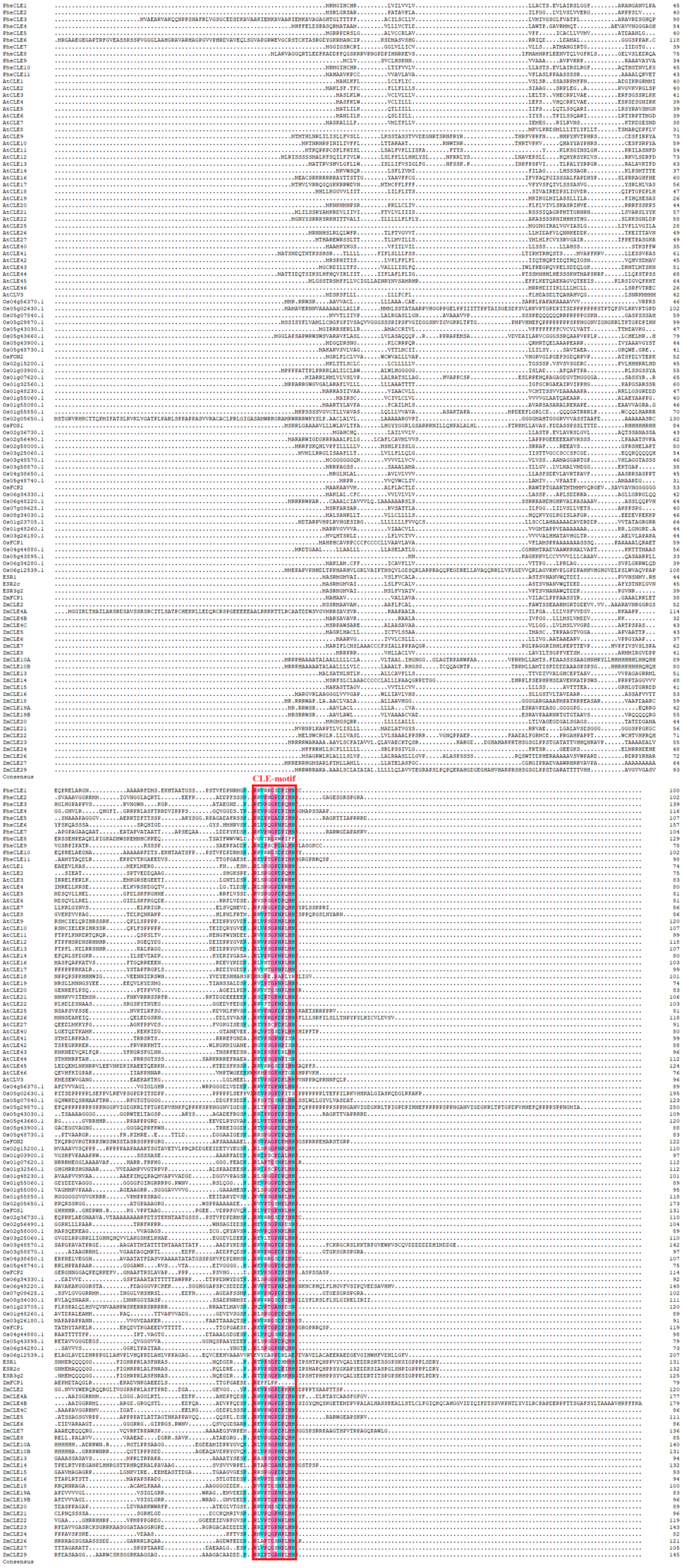
CLE-motif

Supplemental Figure S1. Multiple sequence alignment of CLE families in *P. edulis*, *A. thaliana*, *O. sativa* and *Z. mays*. The left side represents the Gene ID of CLE family members of each species, the middle is the amino acid for multiple sequence alignment, and the number on the right represents the number of amino acids. The red boxes represent the conserved motifs of the CLE families.
