# Supplementary material for: Uncovering PheCLE1 and PheCLE10 Promoting Root Development Based on Genome-Wide Analysis
Source: Int J Mol Sci. 2024 Jun 29;25(13):7190. doi: 10.3390/ijms25137190 (PMC11241622; doi:10.3390/ijms25137190)
Supplement: Supplementary file 1 [file ijms-25-07190-s001.zip › Supplemental Figure S2.pdf]

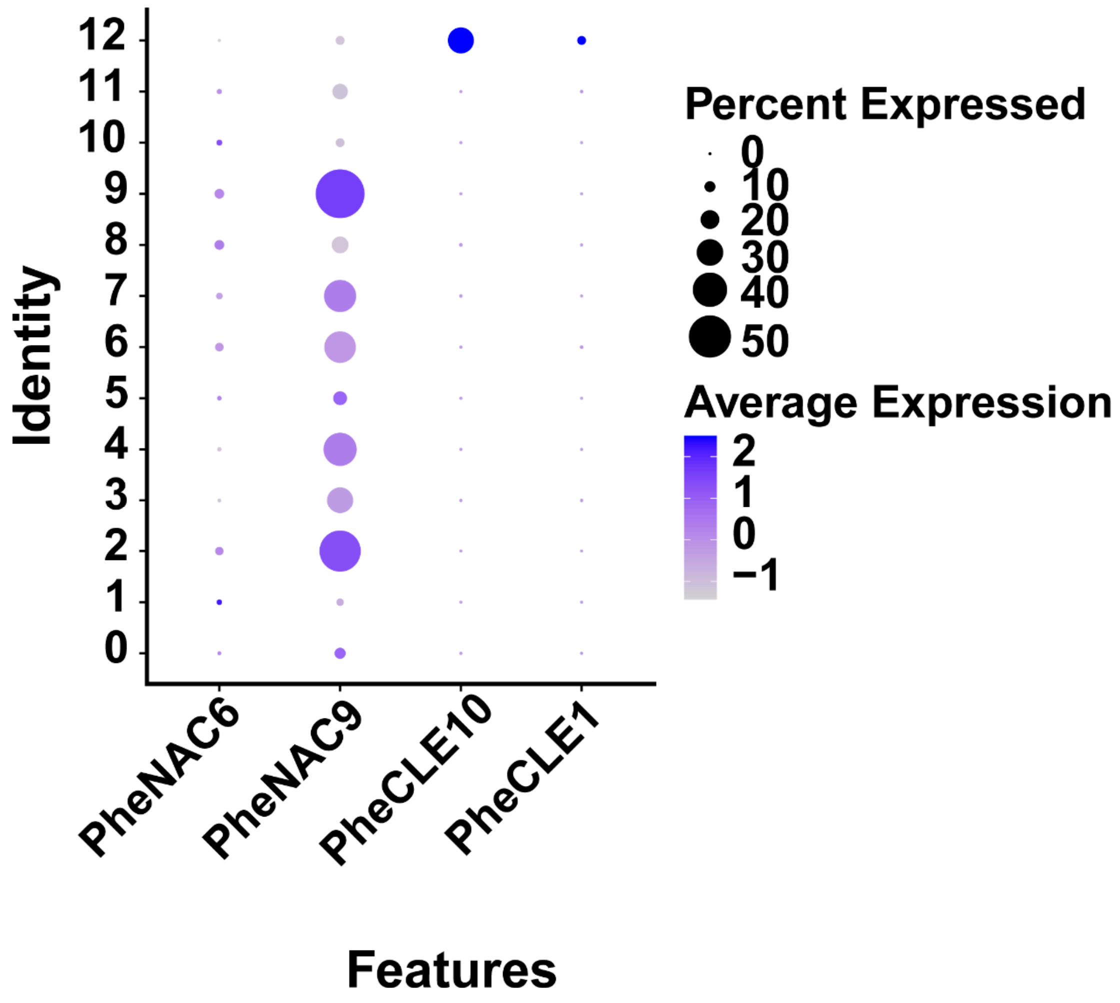

Supplemental Figure S2 The expression patterns of *PheNAC* and *PheCLE* genes in all cell clusters of single cell transcriptome data from the root tips of Moso bamboo basal root.
